# Exploring new therapeutic potentials of curcumin against post-surgical adhesion bands

**DOI:** 10.1186/s12906-022-03808-6

**Published:** 2023-01-31

**Authors:** Mohammad-Mostafa Askarnia-Faal, Sayyed-Hadi Sayyed-Hosseinian, Seyedeh Elnaz Nazari, Fereshteh Asgharzadeh, Ehsan Vahedi, Moein Eskandari, Haniyeh Ghasemi, Amir Avan, Maryam Alaei, Hamideh Naimi, Maryam Daghiani, Atena Soleimani, Abbas Alalikhan, Reza Mohammadzadeh, Gordon Ferns, Mikhail Ryzhikov, Majid Khazaei, Seyed Mahdi Hassanian

**Affiliations:** 1 Department of Clinical Biochemistry, Faculty of Medicine, Mashhad University of Medical Sciences, Mashhad, Iran; 2 Orthopedic Research Center, Shahid Kamyab Hospital, Mashhad University of Medical Sciences, Mashhad, Iran; 3 Department of Medical Physiology, Faculty of Medicine, Mashhad University of Medical Sciences, Mashhad, Iran; 4 Metabolic syndrome Research Center, Mashhad University of Medical Sciences, Mashhad, Iran; 5 Department of Human Genetics, Faculty of Medicine, Mashhad University of Medical Sciences, Mashhad, Iran; 6 Department of Physiotherapy, School of Paramedical Sciences, Mashhad University of Medical Sciences, Mashhad, Iran; 7 Department of Biology, Faculty of Basic Sciences, University of Maragheh, Maragheh, Iran; 8 Division of Medical Education, Brighton & Sussex Medical School, Falmer, Brighton, Sussex BN1 9PH UK; 9 Saint Louis University, School of Medicine, Saint Louis, MO USA

**Keywords:** Phytosomal curcumin, Peritendinous adhesion, Peritoneal fibrosis, Post-surgical adhesion bands, Integrative medicine

## Abstract

**Background:**

Adhesion band formation is a common cause of morbidity for patients undergoing surgeries. Anti-inflammatory and anti-fibrotic properties of curcumin, a pharmacologically active component of *Curcuma longa*, have been investigated in several studies. The aim of this study is to explore the therapeutic potential of curcumin in attenuating post-operative adhesion band (PSAB) formation in both peritoneal and peritendinous surgeries in animal models.

**Methods:**

Bio-mechanical, histological and quantitative evaluation of inflammation, and total fibrosis scores were graded and measured in the presence and absence of phytosomal curcumin.

**Results:**

Results showed that phytosomal curcumin significantly decreased severity, length, density and tolerance of mobility of peritendinous adhesions as well as incidence and severity of abdominal fibrotic bands post-surgery. Curcumin may decrease inflammation by attenuating recruitment of inflammatory cells and regulating oxidant/anti-oxidant balance in post-operative tissue samples. Moreover, markedly lower fibrosis scores were obtained in the adhesive tissues of phytosomal curcumin-treated groups which correlated with a significant decrease in quantity, quality and grading of fibers, and collagen deposition in animal models.

**Conclusion:**

These results suggest that protective effects of phytosomal curcumin against PSAB formation is partially mediated by decreasing inflammation and fibrosis at site of surgery. Further studies are needed to investigate the therapeutic potential of this molecule in preventing PSAB.

## Introduction

Post-operative adhesion bands (PSAB) are fibrotic tissues generated by impaired fibrinolysis and cellular exudates following injury to operated areas such as abdominal cavity [1], flexor and Achilles tendon [2]. Current therapeutic strategy utilizes solid barriers such as Interceed and Seprafilm at reducing adhesion band formation at injury sites. However, accurate prediction of damag levels at injury sites following surgery limits the usage of this method [3, 4]. Peritoneal adhesion bands develop in 93% of patients undergoing a general surgery and up to 97% of patients undergoing an open gynecological pelvic operation [5–7]. The formation of these fibrotic bands is usually asymptomatic [8]. This condition is accompanied by post-surgical complications including pelvic pain, infertility, and intestinal obstruction [9]. Adhesiolysis operations place a significant burden on public health and they are responsible for 3–5% of patient’s death post surgeries. It has been shown that dhesiolysis accounted for 303,836 hospitalizations and more than $1.33 billion in hospitalization and surgeon expenditures [10].

Similarly, peritendinous adhesions are a serious complication of flexor tendon injury associated with a high personal and economic burden for patients [11]. Tendons are dense fibrillary connective tissues made up of parallel collagen fiber bundles and low cellular populations [12]. Flexor tendons serve as energy-saving elastic springs absorbing external forces and stabilizing joint motion and biomechanical function of the musculoskeletal system [13]. Current therapeutic options for reducing or preventing tendon adhesions are ineffective and not routine in clinical medicine. Thus, it is necessary to attain greater understanding of adhesion formation process and develop an effective treatment [12].

Currently, various complementary and alternative medicine methods have been used for human diseases [14, 15]. In line with this, therapeutic potency of many phytochemicals have been investigated to attenuating post-surgical adhesion band formation [15–18]. Curcumin, also known as diferuloylmethane, is a polyphenol extract of turmeric (*Curcuma longa* L. rhizome) and has been used for centuries in traditional Chinese and Indian medicine [19]. Curcumin exerts its therapeutic effects by modulating transcription factors, cell adhesion molecules, enzymes, and cytokines [19]. However, the applicability of curcumin is limited due to low solubility in aqueous mediums, instability at physiological pH, and rapid clearance [20]. To enhance curcumin’s bioavailability and its therapeutic effects, the formulated phytosomal curcumin (curcumin-phosphatidylcholine complex) has been provided by Sami Labs Ltd. (Bangalore, India) and was used in previous publications [21–23].

Aberrant regulation of the inflammatory response and fibrosis are major factors in adhesion band formation [11, 16]. Anti-inflammatory activities of phytosomal curcumin have been reported to have positive effects in various diseases such as cancers [23] and hepatic disorders [24, 25]. In addition, several studies illustrated the protective effects of phytosomal curcumin against liver fibrosis and non-alcoholic fatty liver diseases [26–28]. Previous studies investigating curcumin as a potent anti-inflammatory and antioxidant agent have demonstrated its ability to reduce intra-abdominal adhesion formation induced by dimethyl sulfoxide [29]. In addition, another study showed curcumin improved the quality of tendon rupture healing, which indicates that curcumin holds promise as a treatment for injured tendon tissue [30, 31]. Curcumin is a strong anti-oxidant and anti-inflammatory agent that has different pharmacological effects [20]. In addition, several studies have shown curcumin to be safe at doses up to 8 g per day, however, because of its low solubility in water and rapid metabolism, it does not have high oral bioavailability [20]. Bioavailability of curcumin has been shown to be improved with phytosomal formulations (curcumin complexed with phosphatidylcholine). Phytosomes in complex withphospholipids, exhibit specific physicochemical properties, such as amphiphilicity, allowing them to disperse in hydrophilic and lipophilic media [20]. The efficacy and safety of curcumin phytosomes have not been studied at attenuating post-operative peritoneal and peritendinous adhesions. The aim of this study is to investigate the protective effects of phytosomal curcumin on post-operative peritoneal and peritendinous adhesions.

## Materials and methods

### Materials

Phytosomal curcumin was obtained from Sami Labs Ltd. (Bangalore, India). All reagents for malondialdehyde (MDA), total thiol, catalase (CAT), and superoxide dismutase (SOD) were purchased from Sigma-Aldrich Chemical Co. (St. Louis, MO, USA).

### Animal study

Male Wistar rats (weighing 200–250 g) were obtained from the laboratory animals center of the medical school at Mashhad University of Medical Sciences. Animals were housed according to the protocol approved by Institutional Animal Care Guidelines. All animals had free access to drinking water and were fed standard rat chow. They were kept at a normal temperature of 22–25 °C and a standard 12-hr light/dark cycle.

### Post-operational adhesion band models

General anesthesia was induced with an intraperitoneal injection of ketamine/xylazine. Post-operational peritendinous adhesion model was induced according to a protocol established by Tang et al [32]. Briefly after shaving the right hind limb, a longitudinal incision was made in the Achilles tendon, inducing peritendinous adhesion. The tendon was sutured using the Kessler–Kirchmeyer technique. Abdominal adhesion formation was induced by surgical procedure according to protocol by Hemadeh et al [33]. Briefly, the peritoneal was opened by a U-shaped incision. Using a medical electric scalpel, the cecal and the interior abdomen surfaces were gently rubbed to generate partial petechial hemorrhages and adhesion band formation.

Animals in each model were randomly divided into 3 groups (*n* = 6) as described below: (A) sham group with surgical incision but no adhesion, (B) positive control group with total surgical transection and adhesion receiving normal saline daily, (C) phytosomal curcumin group which is the same as group B except that rats were treated with 25 mg/kg/day curcumin orally [23, 34] for either 7 or 21 days in peritoneal or tendon adhesion models, respectively. At the end of the experiments, rats were anesthetized, sacrificed, and tissue samples collected (rapid freezing by liquid nitrogen or stored in 10% formalin) for further assessments.

### Evaluation of adhesion scores

The macroscopic grading and the severity of the tendon adhesion bands were carried out using the Tang et al. [32] and Ishiyama et al. [35] adhesion scoring system, respectively. The Nair [36] and Leach [37] scoring systems were used for evaluating the incidence and stability of intraperitoneal adhesions, respectively.

### Histological staining

Tissue specimens were fixed in 10% formalin, processed, and embedded in paraffin. Next, tissues were stained with either hematoxylin/eosin (H&E) or Masson’s trichrome staining. H&E staining was performed to analyze general tissue structure and the inflammatory cells infiltration whereas trichrome staining was utilized to explore the collagen deposition, reflecting the severity of fibrosis. Inflammatory cell infiltration was quantified using Moran et al. [38] scoring system. Histological grading scores for the peritendinous adhesion bands were completed according to the Tang et al. system [32].

### Oxidative stress markers analysis

Assessment of the antioxidant effect of phytosomal curcumin was performed by measuring MDA and total thiol concentrations as well as SOD and catalase enzyme activities in tissue samples as described [8, 9].

### Biomechanical testing of tendon repairs

Achilles tendon tissue mechanical properties were analyzed as described previously [39, 40]. In summary, the calcaneus-tendon-muscle complex of rats was dissected and hydrated in phosphate buffered saline (PBS) for 1 hour. Samples were immediately mounted on a tensile testing machine (SANTAM-STM20) using specific metal clamps. The angle between the calcaneus and Achilles tendon corresponded to 30° dorsiflexion of the foot. A 500 N load cell and a 5 mm/min speed were used at a maintained temperature of 25 ± 2 °C. The sample properties were computed using the load-elongation and stress-strain curves obtained during the final load-to-failure tests. The load-elongation curve represents structural parameters including ultimate load (N), elongation (%), energy absorbed, and stiffness. Tendon tissues of the specimens were first tensioned to the point of failure. The maximum longitudinal changes and the maximum load values exerted before tissue rupture are defined as ultimate elongation (mm) and ultimate load (N), respectively [41]. Mechanical data from the stress-stain curve includes ultimate stress (MPa), ultimate strain (%), and tangent modulus (MPa) [40]. Ultimate stress (MPa) is formulated by dividing ultimate load value (N) by cross-sectional area (CSA). Ultimate strain (%) is expressed as elongation rate/initial length (ΔL/L0) × 100. Tangent modulus (MPa) is defined as the ratio of induced stress to strain (the slope of the linear) at each loading cycle, indicating the ability of specimens to resist deformation. Thus, a higher tangent modulus generates higher stress for a given strain [42].

### Statistical analysis

Results were expressed as mean ± standard error of the mean (SEM). Statistical analysis was performed using one-way ANOVA and Shapiro-Wilk normality test. A *P*-value of < 0.05 was considered significant. All statistical assessments were performed using the SPSS software (SPSS Inc., Chicago, IL, USA).

## Results

### Phytosomal curcumin attenuates frequency and structural properties of adhesion bands

**Fig. 1:**
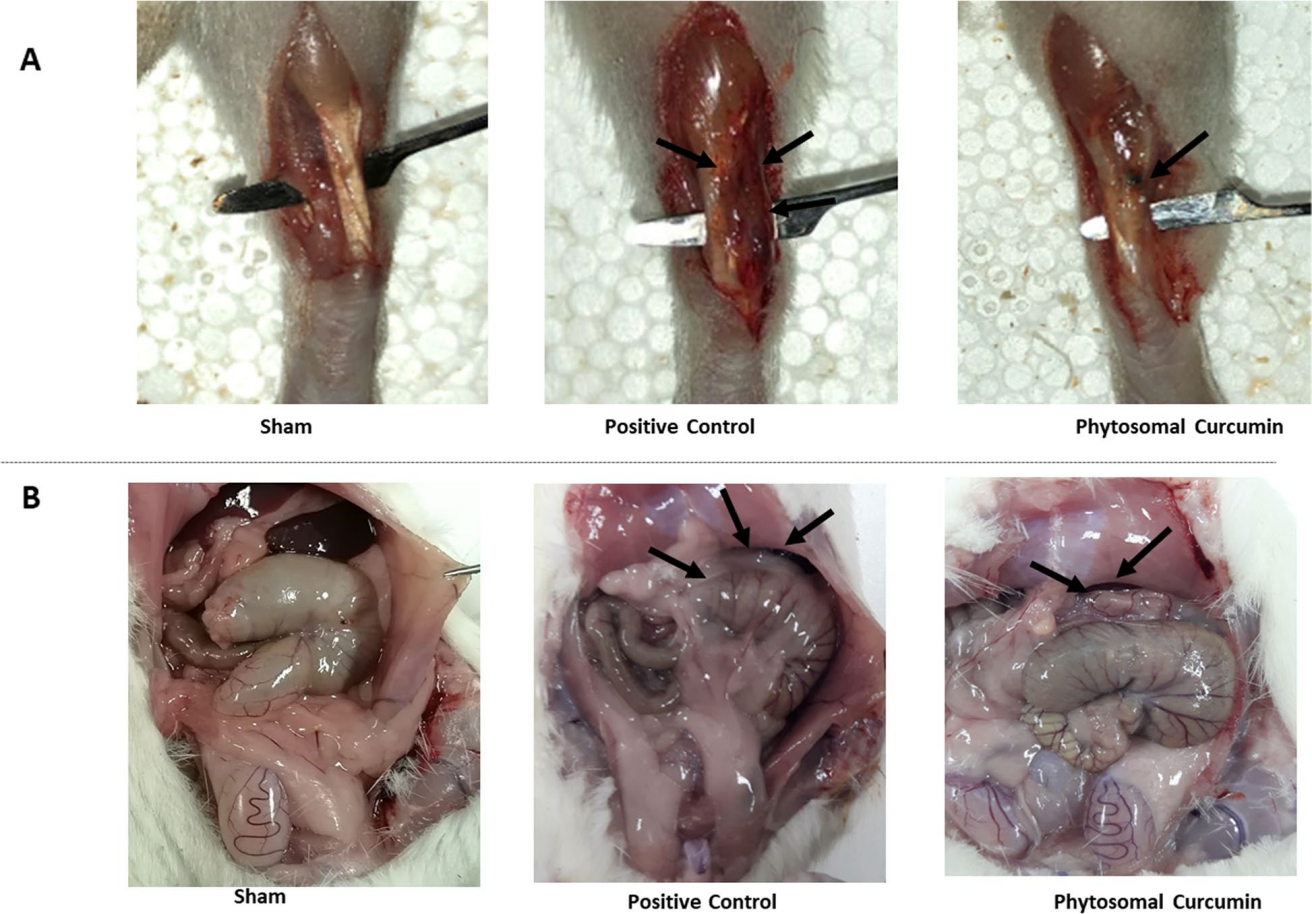
Phytosomal curcumin attenuated the formation of adhesion bands. **a-b** The macroscopic illustration of adhesion bands formation in different groups of peritendinous (**a**), and peritoneal (**b**) post-surgical adhesion models

Anesthetic induction and surgical procedures were successful with all rats surviving to the end of the study. Results showed that curcumin significantly decreased adhesion band formation in both the tendon (Fig. 1A) and abdominal surgeries (Fig. 1B). Tang [32] and Ishiyama grading [35] system were used to evaluate the properties and severity of peritendinous adhesions, and Nair [36] and Leach [37] scoring systems for evaluating the presence and rigidity of peritoneal adhesions.

**Fig. 2:**
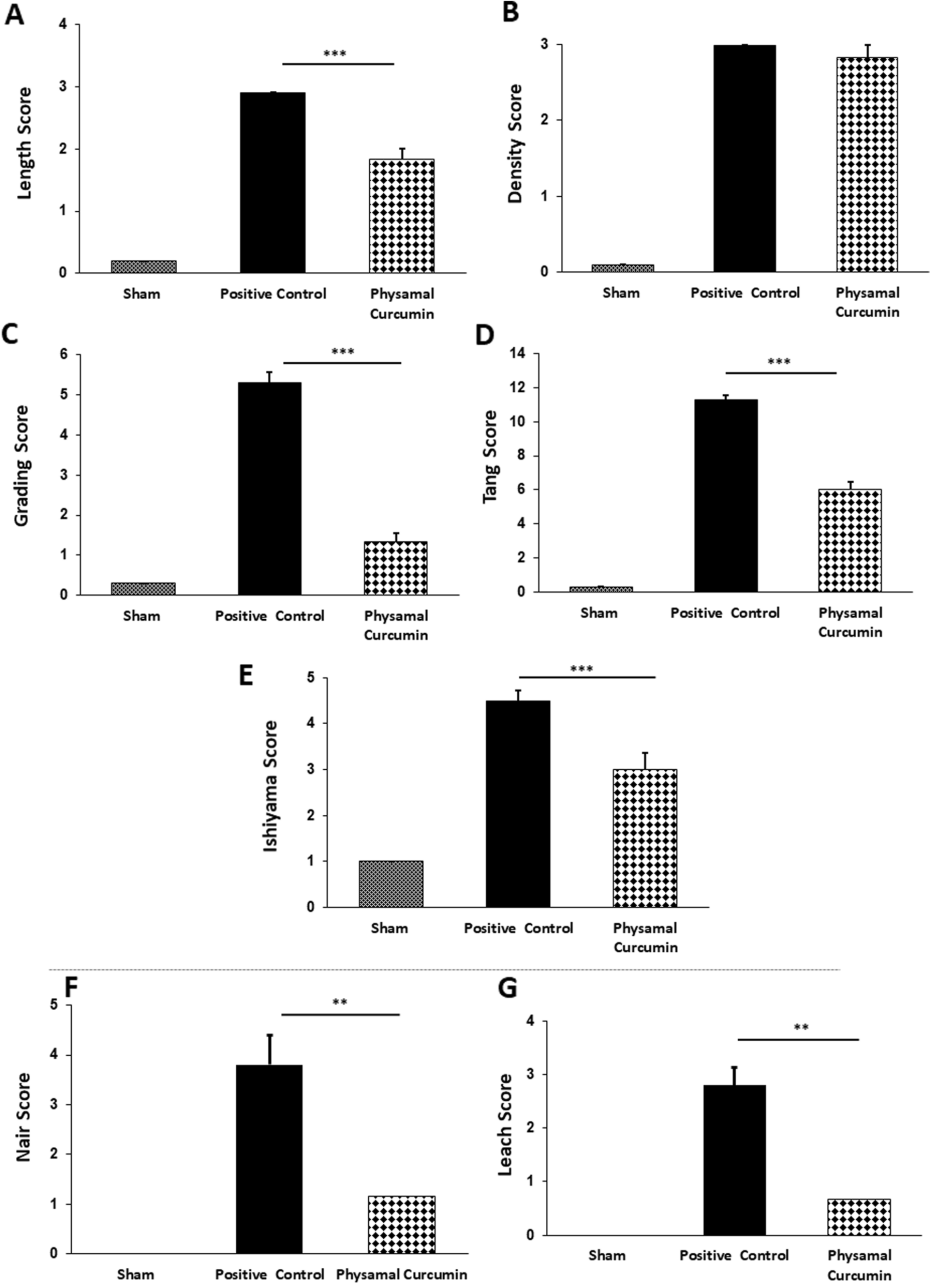
Phytosomal curcumin attenuates macroscopic grading of post-operational adhesion bands score. **a-d** The macroscopic adhesion grading based on Tang et al. scoring system [32] for peritendinous adhesion bands. **e** The same as (A-D) except that severity of adhesion bands was scored according to Ishiyama et al. grading system [35]. **f-g** The efficacy of phytosomal curcumin on reduction of Nair [36] (**f**) and Leach [37] (**g**) grading systems in abdominal post-surgery. **P < 0.01, ***P < 0.001

Using Tang macroscopic grading scores of peritendinous adhesion bands [32], curcumin-treated rats showed decreased length (Fig. 2A), density and tolerance for mobility (Fig. 2B), and grading of adhesion score (Fig. 2C) compared to the positive control group. Consistent with these findings, the Tang and Ishiyama macroscopic scoring system [32, 35] also indicated that curcumin treatment decreased the severity of adhesions formation (Fig. 2D and E).

Next, Nair [36] and Leach scoring systems [37] were used to investigate the protective effects of curcumin on abdominal adhesion bands formation. Results showed that curcumin significantly decreased both the incidence (Fig. 2F) and severity (Fig. 2G) of fibrotic bands compared to positive control rats. No adhesion fibers were found in the sham group.

### Phytosomal curcumin inhibits post-surgical inflammation in peritendinous and intraperitoneal adhesion models

**Fig. 3:**
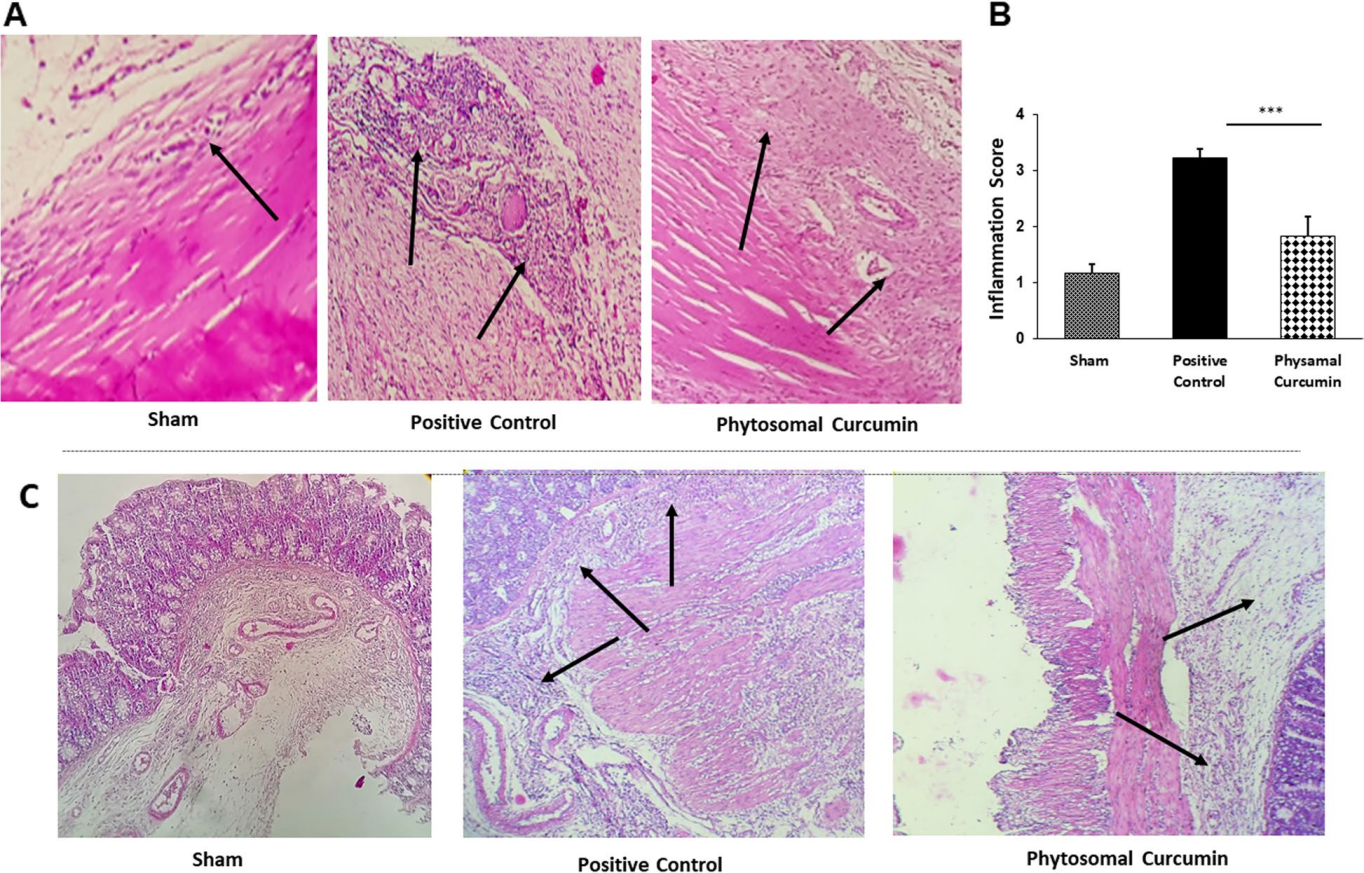
Phytosomal curcumin reduced inflammatory cells infiltration to the site of surgeries. **a** Hematoxylin and Eosin (H&E) staining of Achilles tendon adhesion tissues showed a lower leukocyte infiltration (arrows) into the tendon tissue in phytosomal curcumin-treated group. **b** Quantification of inflammation score based on Moran et al. scoring system [38]. **c** The effect of phytosomal curcumin was also compared between abdominal adhesion groups, using H&E staining. Arrows indicate inflammatory cells infiltration ***P < 0.001

Inflammation is one of the key factors in the pathogenesis of post-surgical adhesion band formation. To determine the protective effect of phytosomal curcumin on adhesion band-associated inflammation, either tendon or abdominal adhesion tissues were stained with H&E to examine the morphological and histological changes in adhesion rat models. It has been suggested that 25 mg/Kg phytosomal curcumin has potential therapeutic effects [22, 23]. Results showed that using the same dose of curcumin potently decreased infiltration of inflammatory cells into the injury site (Fig. 3A) and decreased fibrosis in the tendon adhesions (Fig. 3B). In terms of peritoneal adhesions, H&E results also demonstrated a decrease in adhesion-related inflammation based on a lower influx of inflammatory cells into the surgical area (Fig. 3C).

### Phytosomal curcumin suppresses inflammation by counterbalancing oxidative stress

**Fig. 4:**
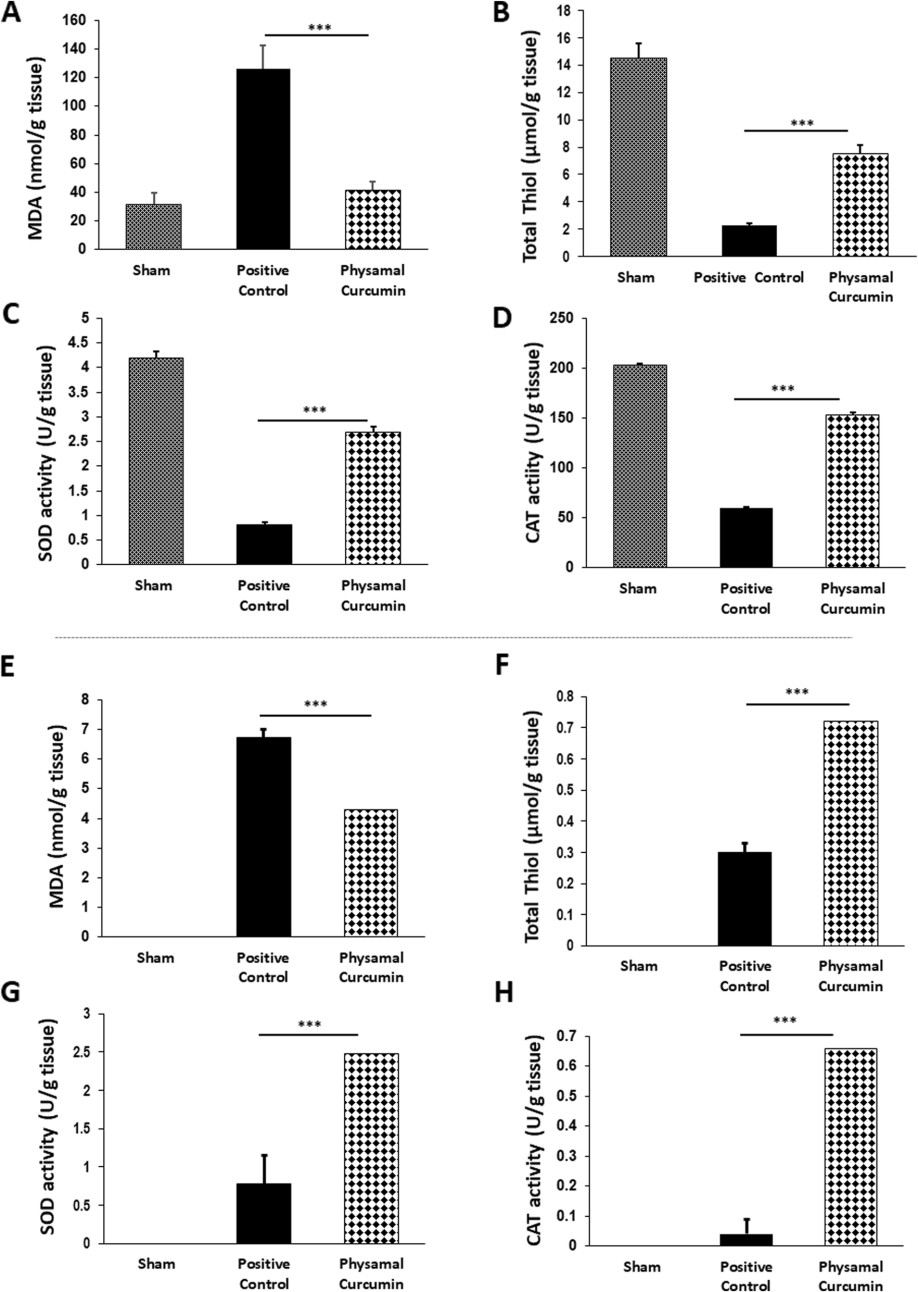
The effect of phytosomal curcumin on oxidant/ anti-oxidant balance in adhesive tissue samples. **a-d** The concentration of MDA (**a**) and total thiol (**b**), as well as superoxide dismutase (**c**) and catalase (**d**) enzyme activities, were compared between different groups in peritendinous adhesions. **e-h** The protective effect of phytosomal curcumin on counterbalancing of oxidative stress was performed by measuring oxidative stress markers in abdominal tissue homogenates. ***P < 0.001

Evaluation of oxidative stress markers was used to further investigate the anti-inflammatory activity of curcumin in post-surgical adhesion band models. Compared to the positive control group, curcumin treatment significantly reduced the level of MDA, an oxidative marker of fatty acid peroxidation, in both peritendinous (Fig. 4A) and abdominal adhesion tissue homogenates (Fig. 4E).

Next, total thiol (Fig. 4B, and F) concentration and activities of SOD (Fig. 4C, and G) and CAT (Fig. 4D, and H) enzymes, all anti-oxidant markers, were measured in the Achilles tendon and abdominal adhesion tissues. These results clearly showed that the level and activity of all these anti-oxidant markers was increased in the curcumin-treated group, supporting the anti-inflammatory effects of phytosomal curcumin in post-surgical adhesion rat models.

### The effects of curcumin on fibrosis as a key element in post-surgical adhesion band formation

**Fig. 5:**
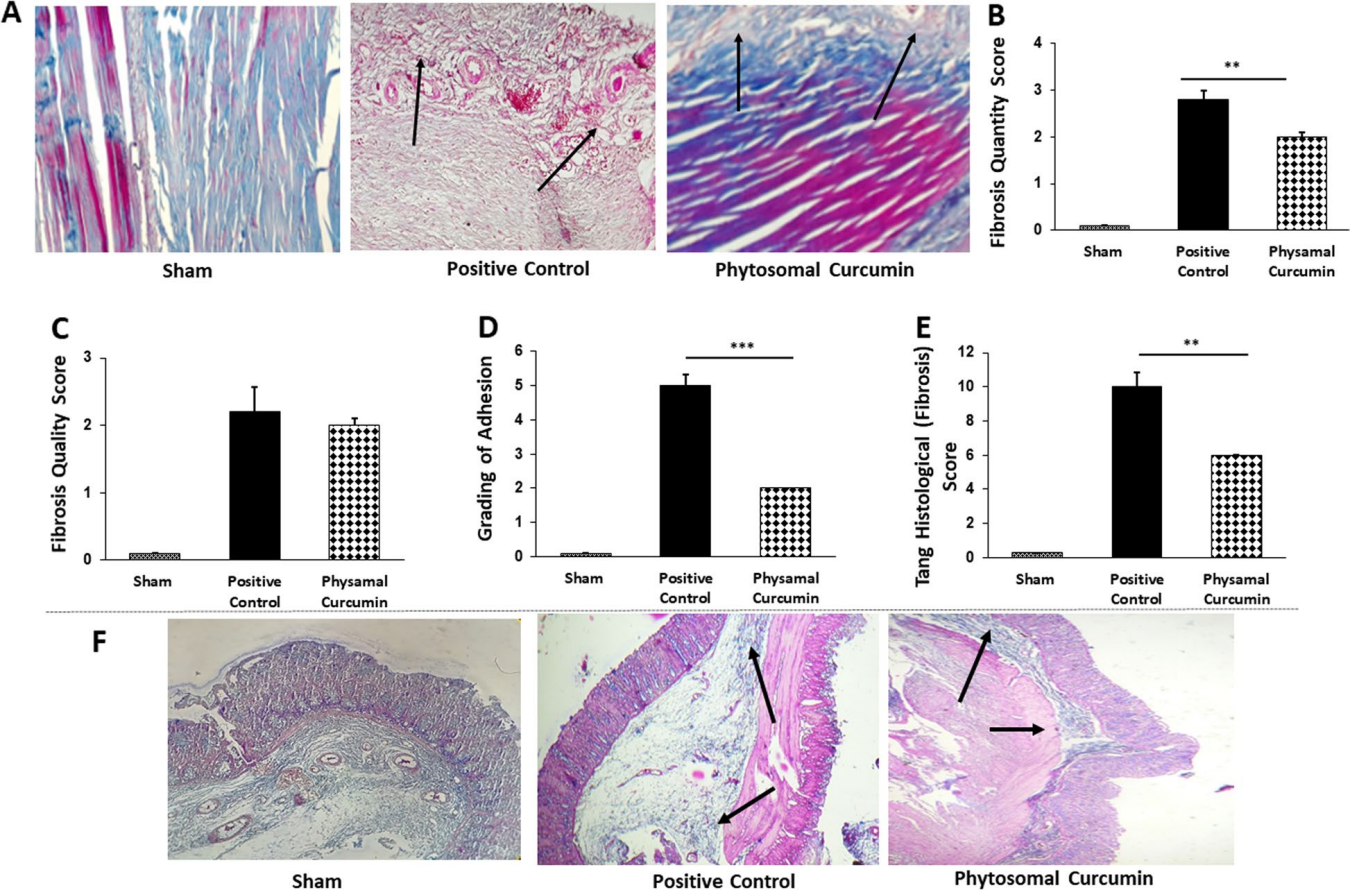
Phytosomal curcumin decreased post-surgical fibrosis in animal models. **a** Results of Masson’s trichrome staining showed a significant reduction of collagen deposition in curcumin-treated rats compared to the positive control group in tendon adhesion tissues. **b-e** Tang Histological (microscopic) grading score [32] (**e**), consisting of quantity (**b**), quality (**c**), and grading (**d**) of fibrosis, was compared between different groups in post-operational peritendinous adhesion band formation. **f** Masson’s trichrome staining showed that phytosomal curcumin attenuated collagen deposition in post-surgical peritoneal adhesions. Arrows show deposition of collagen. **P < 0.01, ***P < 0.001

Tissues were stained with Masson’s trichrome to determine the effect of curcumin on fibrosis and collagen deposition at the surgery site. Results showed that compared to the positive control rats, curcumin suppressed fibrosis and collagen content in peritendinous adhesion tissues (Fig. 5A). Moreover, by using Tang Histological (microscopic) grading score [32] (Fig. 5E), consisting of quantity (Fig. 5B), quality (Fig. 5C), and grading (Fig. 5D) of fibrosis, showed that curcumin significantly decreased the overall fibrosis score when compared with the positive control group in peritendinous adhesion. Similarly, using Masson’s trichrome staining, the efficacy of phytosomal curcumin on fibrosis of peritoneal tissues was investigated and results showed potent protective activities of curcumin against fibrosis at surgery site (Fig. 5F). These results suggest that inhibition of fibrosis is a mechanism by which curcumin decreases post-surgery adhesion band formation.

### Effects of phytosomal curcumin on mechanical properties of tendons

To ascertain the effects of phytosomal curcumin on structural and mechanical properties of tendon adhesion tissues, the load-elongation and the stress-strain curves were plotted. Cross-sectional area (CSA) was also measured to evaluate possible modifications in mechanical features of the Achilles tendon in response to phytosomal curcumin. Results showed that in comparison to the positive control group, phytosomal curcumin improved the structural properties of damaged tissues, including maximum load (N) in tendons (Table 1). However, these results were not statistically significant.

**Table 1:** Comparing structural/mechanical properties of the Achilles tendon between groups. Values are expressed as means ± standard deviation

Group	Ultimate Load (N)	Ultimate Stress (MPa)	Ultimate Strain (%)	Tangent Modulus (MPa)
Sham	40.6 ± 5.126	12.92 ± 1.632	34.56 ± 9.621	54.19 ± 18.74
Positive Control	35.5 ± 4.083	2.232 ± 0.256	40.94 ± 16.4	6.694 ± 2.89
Phy-curcumin	41.55 ± 5.541	3.663 ± 0.488	40.05 ± 20.58	8.487 ± 5.493

Since the load-elongation curve depends on size, volume, and shape of tissue samples, the quantities were normalized by using the stress-strain curve analyzing the ultimate stress (MPa), ultimate strain (%), and tangent modulus (MPa) indexes. As expected, in the sham group, the level of ultimate stress and ultimate strain is higher and lower than positive control group, respectively. Moreover, phytosomal curcumin improved ultimate stress and strain when compared to the positive control group. Consistently, compared to the positive control group, treatment with phytosomal curcumin increased tangent modulus which is an index indicating the ability of specimens to resist deformation (Table 1).

## Discussion

In this study the protective effects of oral phytosomal curcumin in decreasing adhesion formation post tendon and abdominal surgeries were investigated in animal models. Results suggested that phytosomal curcumin significantly decreased post-operational adhesion band formation in both rat models. Moreover, phytosomal curcumin reduced adhesion-related inflammatory responses by decreasing infiltration of inflammatory cells and regulating the oxidant/anti-oxidant balance at surgery sites. Furthermore, phytosomal curcumin potently exhibited anti-fibrotic activities by attenuating fibrotic bundle thickness and collagen deposition. These findings support the therapeutic potential of phytosomal curcumin in decreasing post-surgical adhesion band formation.

Adhesion band formation post tendon and abdominal injuries are common surgery-associated complications in patients worldwide [43–46]. Inflammation is a key physio-pathological factor in post-surgical adhesion band formation [47, 48]. The anti-inflammatory properties of phytosomal curcumin and its safety have been validated in numerous human disorders including osteoarthritis, diabetes, cancer, retinopathy, and other diseases [49–51]. It has been shown that curcumin down-regulates expression of several inflammatory mediators including IL-6, TNF-α, nuclear factor kappa-B (NF-κB)-regulated gene products such as cyclooxygenase (COX)-2, IL-1, cell adhesion molecules, and C-reactive protein (CRP) [19]. Similarly, Vizzutti et al. showed that production of reactive oxygen species (ROS) was reduced in curcumin-treated mice in a steato-hepatitis model [52]. It has been recently shown that the anti-cancer property of phytosomal curcumin is partially mediated by eliciting anti-inflammatory responses in colorectal cancer [22]. Consistent with these findings Marjaneh et al. showed that phytosomal curcumin potentiates the anti-inflammatory activity of 5-fluorouracil (5-FU), leading to a significant reduction in inflammation and histo-pathological scores in colitis-associated colorectal cancer using in vitro and in vivo models [23]. In another study we demonstrated the anti-oxidant activities of phytosomal curcumin in a xenograft mice model of breast cancer [21]. Consistent with these findings, results of this study showed that curcumin elicits significant anti-inflammatory activity by decreasing inflammatory cell infiltration and increasing levels and activities of anti-oxidant markers in both peritendinous and abdominal surgeries. These results suggest that a decreased inflammatory response post-surgery could be a mechanism by which curcumin elicits its therapeutic potency at injury sites.

Although surgically-induced adhesions and inflammatory responses occur early during the adhesions formations, fibrosis appears as a late event with a major impact on tissues dysfunction [11, 16]. In line with this, Kang et al. evaluated the protective effects of curcumin on synthesis of collagen in both cellular and animal models. Results showed a lower thickness of smooth muscle alpha-actin and collagen fibers and lower mRNA expression of type I collagen in curcumin-treated groups [53]. Furthermore, it has been shown that the high density of fibrillar extracellular matrix (ECM) and the gene expression levels of pro-collagen type I were reduced via curcumin treatment inhibiting fibrogenic progression in sinusoids and perivenular areas in steatohepatitis mice [52]. Consistently, results of this study showed that curcumin via reduction of fibrosis quantity, fibrosis quality, grading of adhesion, and collagen deposition could decrease total fibrosis score in tissue adhesions in rat model.

These results suggest that protective response of phytosomal curcumin against PSAB formation is partially mediated by decreasing inflammation and fibrosis at site of surgery. Further studies are needed to investigate therapeutic potential of this molecule in preventing PSAB.”. The exact protective mechanisms of phytosomal curcumin in adhesion models have yet to be understood. Further animal and clinical studies are needed to clarify these underlying mechanisms and validate these results in patients.

## Conclusion

Results of this study suggest that the protective response of phytosomal curcumin against PSAB formation is partially mediated by decreasing inflammation and fibrosis at surgery sites. Supplementary animal and clinical studies are required to elucidate these underlying mechanisms and confirm these results in patients.

## Data Availability

All data and materials are available upon request to corresponding author via sending e-mail to hasanianmehrm@mums.ac.ir.

## Abbreviations

PSAB: Post-operative adhesion bands
MDA: Malondialdehyde
CAT: Catalase
SOD: Superoxide dismutase
H&E: Hematoxylin/eosin
PBS: Phosphate buffered saline
SEM: Standard error of mean
IL-1: Interlukine-1
IL-6: Interlukine-6
TNF-α: Tumor necrosis factor Alpha
NF-κB: Nuclear factor kappa B
COX-2: Cyclooxygenase-2
CRP: C-reactive protein
ROS: Reactive oxygen species
5-FU: 5 Fluorouracil
